# Supplementary material for: Anaplastic Lymphoma Kinase (ALK) Inhibitors Enhance Phagocytosis Induced by CD47 Blockade in Sensitive and Resistant ALK-Driven Malignancies
Source: Biomedicines. 2024 Dec 12;12(12):2819. doi: 10.3390/biomedicines12122819 (PMC11673128; doi:10.3390/biomedicines12122819)
Supplement: Supplementary file 1 [file biomedicines-12-02819-s001.zip › biomedicines-3247478-supplementary.pdf]

Supplementary Materials

ALK inhibitors enhance phagocytosis induced by CD47 blockade in sensitive and resistant ALK-driven malignancies

Federica Malighetti, Matteo Villa, Mario Mauri, Simone Piane, Valentina Crippa, Ilaria Crespiatico, Federica Cocito, Elisa Bossi, Carolina Steidl, Ivan Civettini, Chiara Scollo, Daniele Ramazzotti, Carlo Gambacorti-Passerini, Rocco Piazza, Luca Mologni and Andrea Aroldi

Supplementary Tables

Table S1. List of ALK-positive cancer cell lines used and the corresponding mechanism of resistance to Tyrosine Kinase Inhibitors (TKIs) if present.

Cell line	Cancer type	ALK mutation	ALK-independent mechanism of resistance
**SUP-M2**	ALCL	WT	/
**Karpas-299 (K299)**	ALCL	WT	/
**AS4**	ALCL	L1196M (resistance to crizotinib)	RAS/MAPK and PI3K/AKT pathways activation (resistance to lorlatinib)
**CLB-Ga**	Neuroblastoma	R1275Q (resistance to crizotinib)	/
**CLB-Ga-LR1000**	Neuroblastoma	R1275Q (resistance to crizotinib)	ErbB family and MAPK pathways activation (resistance to lorlatinib)
**H3122**	Lung adenocarcinoma	WT	/
**H3122-LR100**	Lung adenocarcinoma	WT	EGFR activation (resistance to lorlatinib)

Abbreviations: ALCL, Anaplastic Large T-Cell Lymphoma; WT, wild type; RAS/MAPK, Mitogen-Activated Protein Kinase; PI3K/AKT, Phosphatidylinositol 3-Kinase/AKT; ErbB, Erythroblastic oncogene B; EGFR, Epidermal Growth Factor Receptor.

Table S2. Antibodies used with corresponding clone, purpose and company (Flow Cytometry, FC).

Antibody	Clone	Purpose	Company
**Anti-human Calreticulin (CALR)**	FMC75 (conjugated)	FC	Abcam
**Anti-human CD47**	REA220 (conjugated)	FC	Miltenyi Biotec
	B6H12.2 (unconjugated)	Treatment	BioXCell
**Human IgG_1 isotype control**	N/A (Cat. # BE0297)	Treatment	BioXCell
**Anti-human CD11b**	REA713 (conjugated)	FC	Miltenyi Biotec
**Anti-human CD14**	REA599 (conjugated)	FC	Miltenyi Biotec
**Anti-human SIRP-α**	REA144 (conjugated)	FC	Miltenyi Biotec

Supplementary Figures

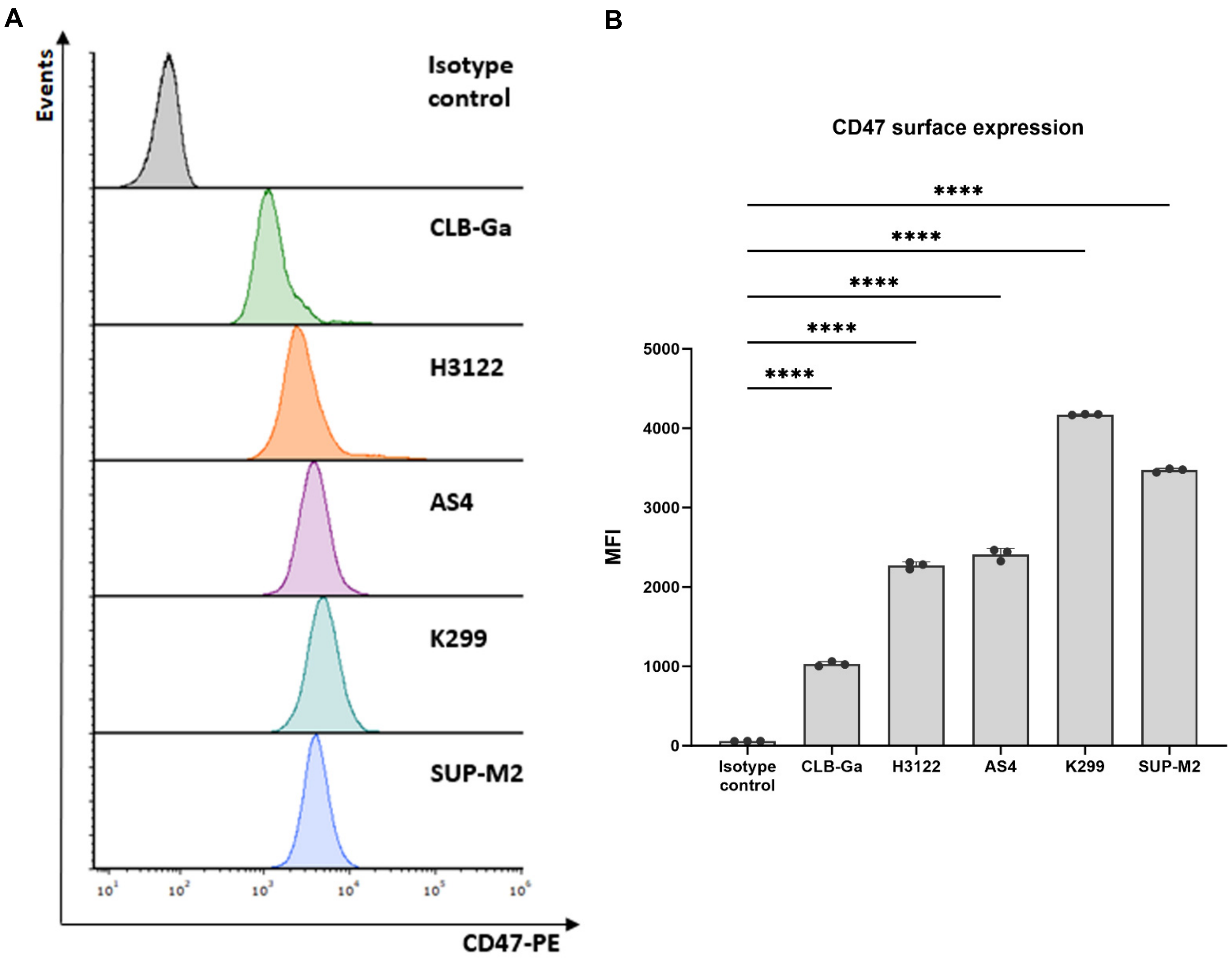

Figure S1

Figure S1

Surface expression of CD47 in ALK-positive cancer cell lines

Representative histogram (A) and Median Fluorescence Intensity (MFI, B), by flow cytometry, of CD47 expression in a panel of ALK-positive cancer cell lines (Neuroblastoma: CLB-Ga; Non-small Cell Lung Cancer (NSCLC): H3122; Anaplastic Large cell Lymphoma: SUPM-2, K299, AS4) compared to isotype control (one-way ANOVA with multiple comparisons correction; CD47 $F_{(5,12)} = 4670$; experimental triplicate; **** $p < 0.0001$).

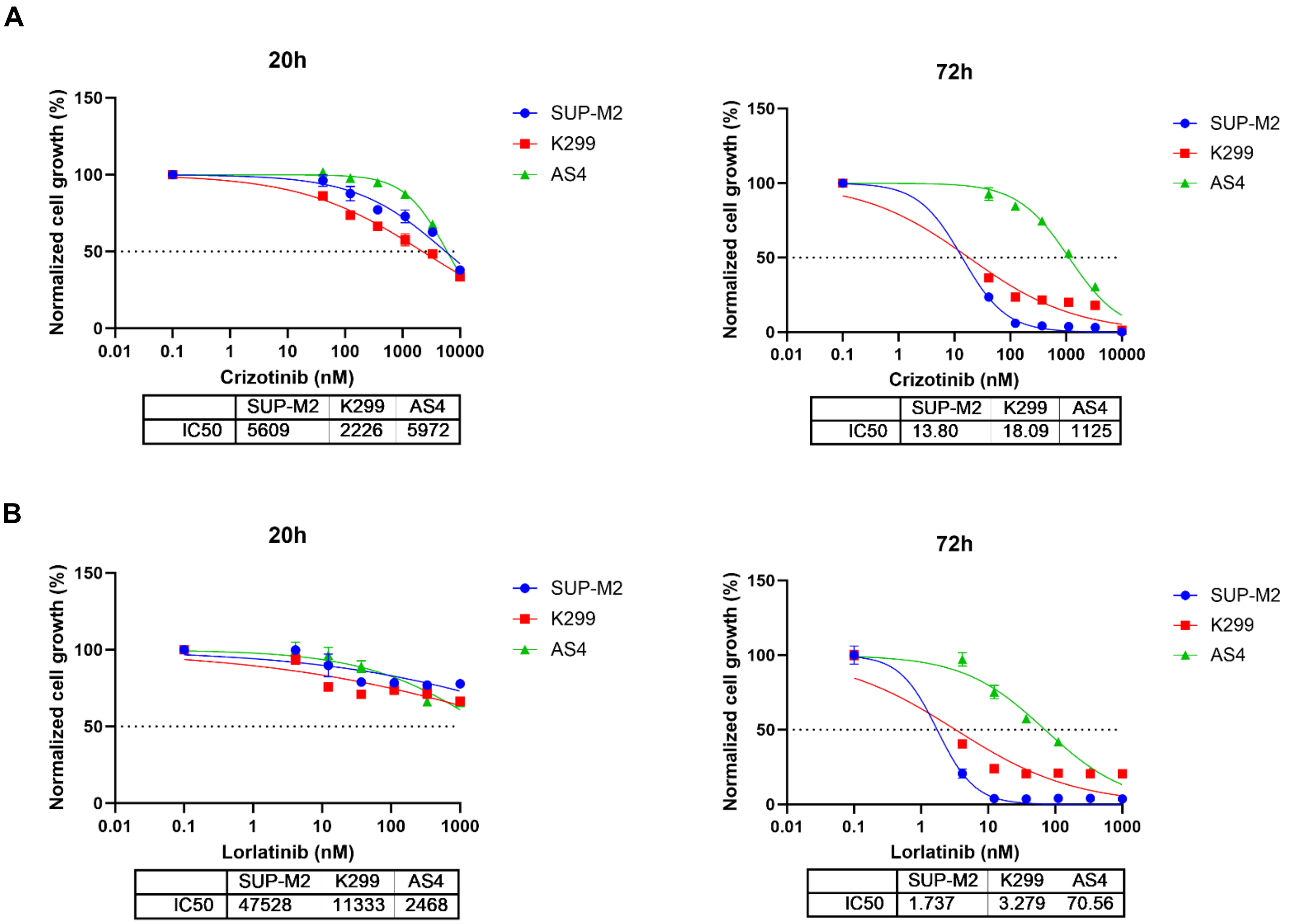

	SUP-M2	K299	AS4
IC50	5609	2226	5972

	SUP-M2	K299	AS4
IC50	13.80	18.09	1125

	SUP-M2	K299	AS4
IC50	47528	11333	2468

	SUP-M2	K299	AS4
IC50	1.737	3.279	70.56

Figure S2

Figure S2

Cell proliferation assay in ALK-driven lymphoma cell lines (SUP-M2, K299, AS4) after exposure to increasing concentration of crizotinib or lorlatinib at different timepoints (20 and 72 hours). A-B) Absolute IC_{50} values showed that inhibitory effects on cell growth were obtained at much higher concentrations of TKIs than those used for CALR upregulation and co-culture assays after 20 hours TKIs exposure. IC_{50} values for each cell lines are outlined under each cell proliferation graph.

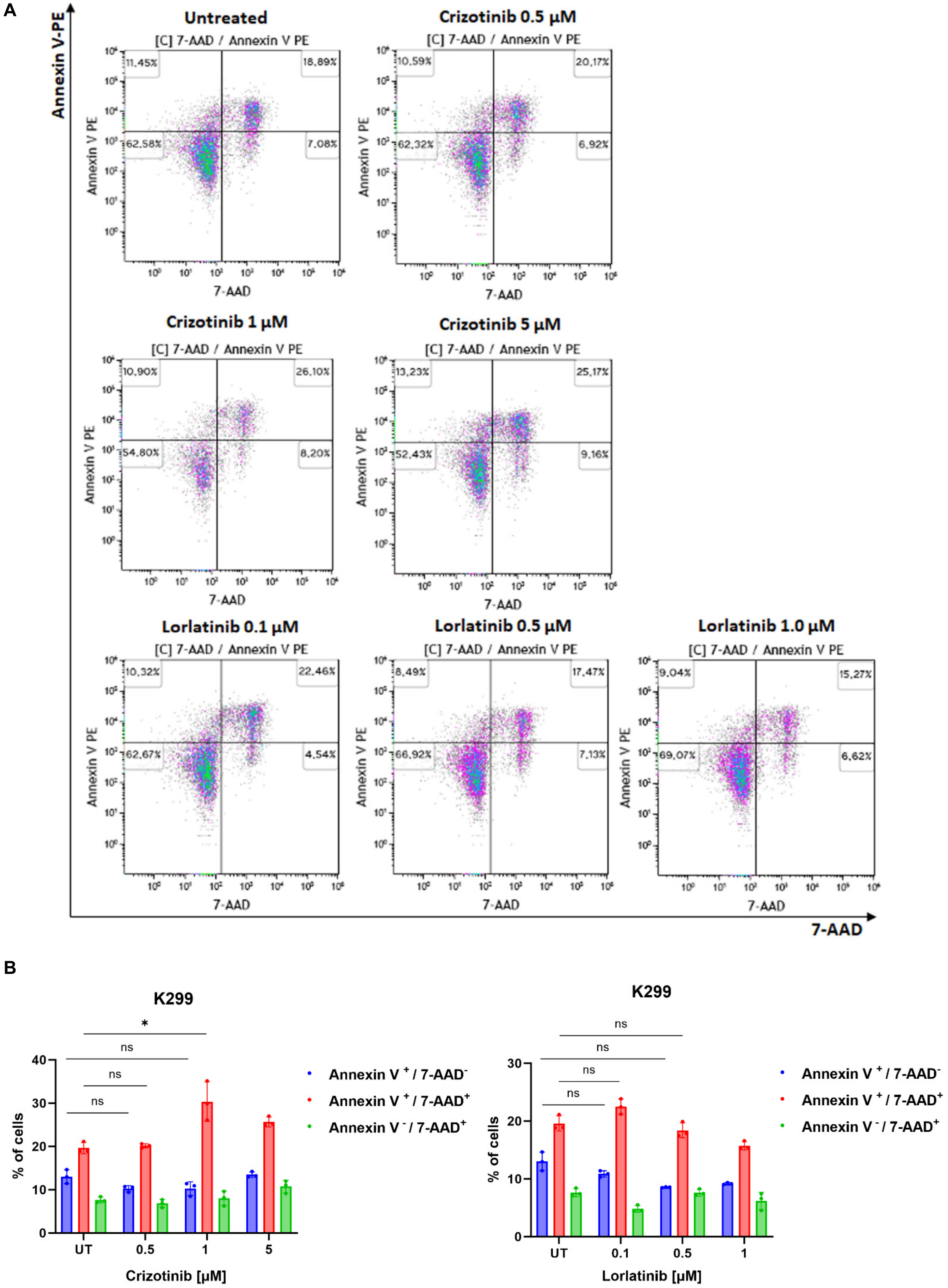
A
Annexin V-PE
7-AAD
Untreated
Crizotinib 0.5 µM
Crizotinib 1 µM
Crizotinib 5 µM
Lorlatinib 0.1 µM
Lorlatinib 0.5 µM
Lorlatinib 1.0 µM
[C] 7-AAD / Annexin V PE
11,45% 18,89% 62,58% 7,08%
10,59% 20,17% 62,32% 6,92%
10,90% 26,10% 54,80% 8,20%
13,23% 25,17% 52,43% 9,16%
10,32% 22,46% 62,67% 4,54%
8,49% 17,47% 66,92% 7,13%
9,04% 15,27% 69,07% 6,62%
B
K299
% of cells
Crizotinib [µM]
UT 0.5 1 5
Lorlatinib [µM]
UT 0.1 0.5 1
ns
*
Annexin V + / 7-AAD-
Annexin V + / 7-AAD+
Annexin V - / 7-AAD+

Figure S3

Figure S3

Annexin V assay as approach to assess entity of dead and apoptotic cells after treatment of K299 with TKIs for 20 hours. A-B) Representative flow cytometry plots, after Annexin V and 7-AAD staining, as quantification of dead cells (Annexin V^+/7-AAD^+) and apoptotic cells (Annexin V^+/7-AAD^-) in ALK-positive lymphoma K299 cell line. Exposure of K299 to crizotinib (0.5 μM) or Lorlatinib (100 nM) for 20 hours, resulting in upregulation of CALR as shown, did not correlate with an increase in dead cells or apoptotic cells if compared to the untreated (UT) condition (unpaired, two-tailed Student's *t*-test. Dead.$K299_{CRIZO}$: UT vs. 0.5 μM, not significant (ns); UT vs. 1.0 μM, * $p < 0.05$. Apoptotic.$K299_{CRIZO}$: UT vs. 0.5 μM, ns; UT vs. 1.0 μM, ns. Dead.$K299_{LORLA}$: UT vs. 0.1 μM, ns; UT vs. 0.5 μM, ns. Apoptotic.$K299_{LORLA}$: UT vs. 0.1 μM, ns; UT vs. 0.5 μM, ns).

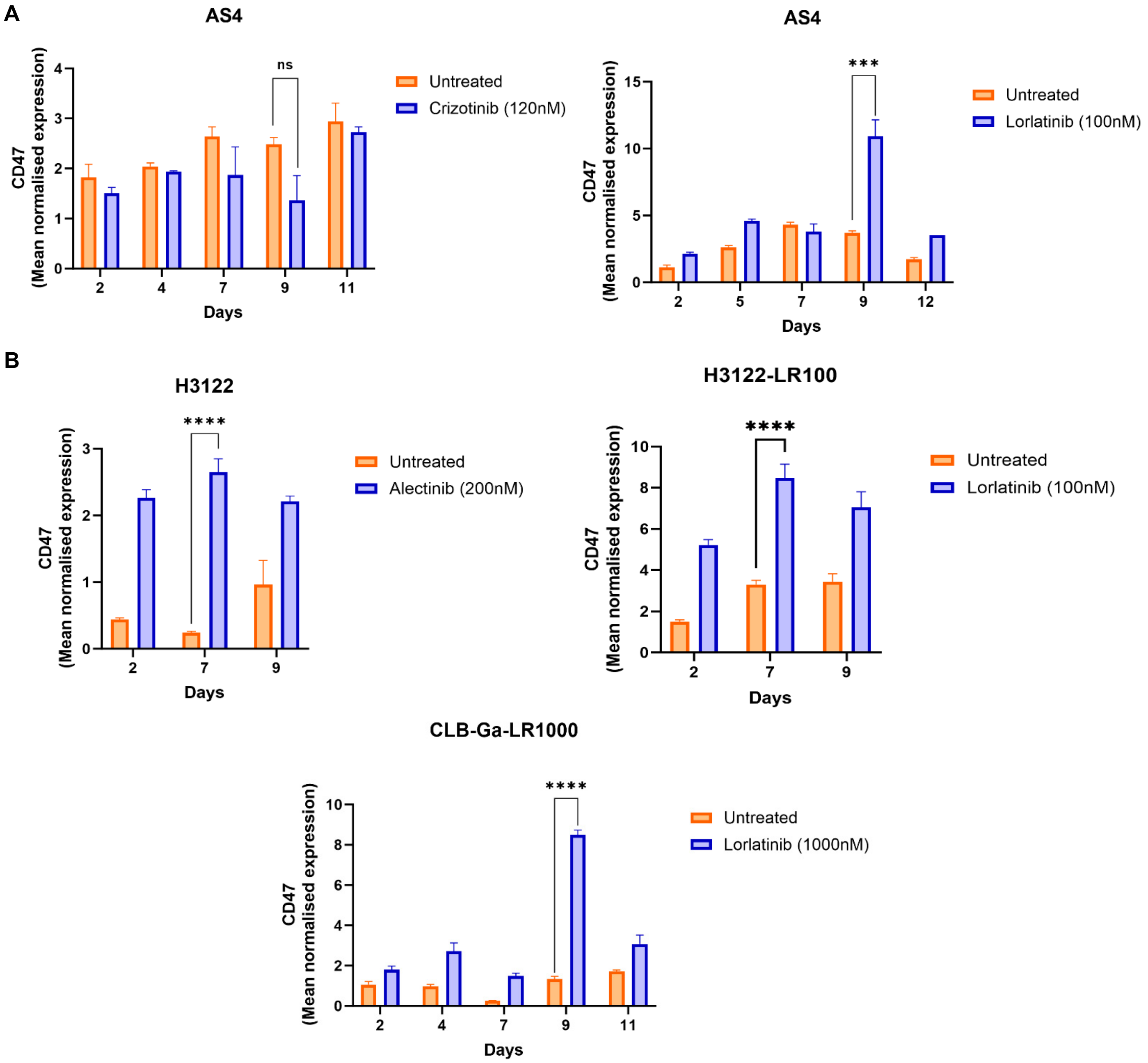

Figure S4

CD47 RNA expression in ALK-positive cell lines after exposure to TKIs at different timepoints. A-B) Representative histogram bars of CD47 RNA expression, by real time quantitative PCR, showed that crizotinib did not induce increased levels of CD47 RNA in AS4 cell lines, whereas lorlatinib promoted overexpression of CD47 RNA by day +9 if compared to untreated control (A). H3122 (both sensitive to alectinib and resistant to lorlatinib) experienced CD47 RNA overproduction by day +7 and, similarly to AS4, resistant CLB-Ga-LR1000 showed higher levels of CD47 RNA than untreated control (unpaired, one-tailed Student's *t*-test. Day9.AS4$_{CRIZO}$: UT vs. 120 nM, not significant (ns). Day9.AS4$_{LORLA}$: UT vs. 100 nM, *** $p < 0.001$. Day7.H3122$_{ALECTINIB}$: UT vs. 200 nM, **** $p < 0.0001$. Day7.H3122-LR100$_{LORLA}$: UT vs. 100 nM, **** $p < 0.0001$. Day9.CLB-Ga-LR1000$_{LORLA}$: UT vs. 1000 nM, **** $p < 0.0001$).

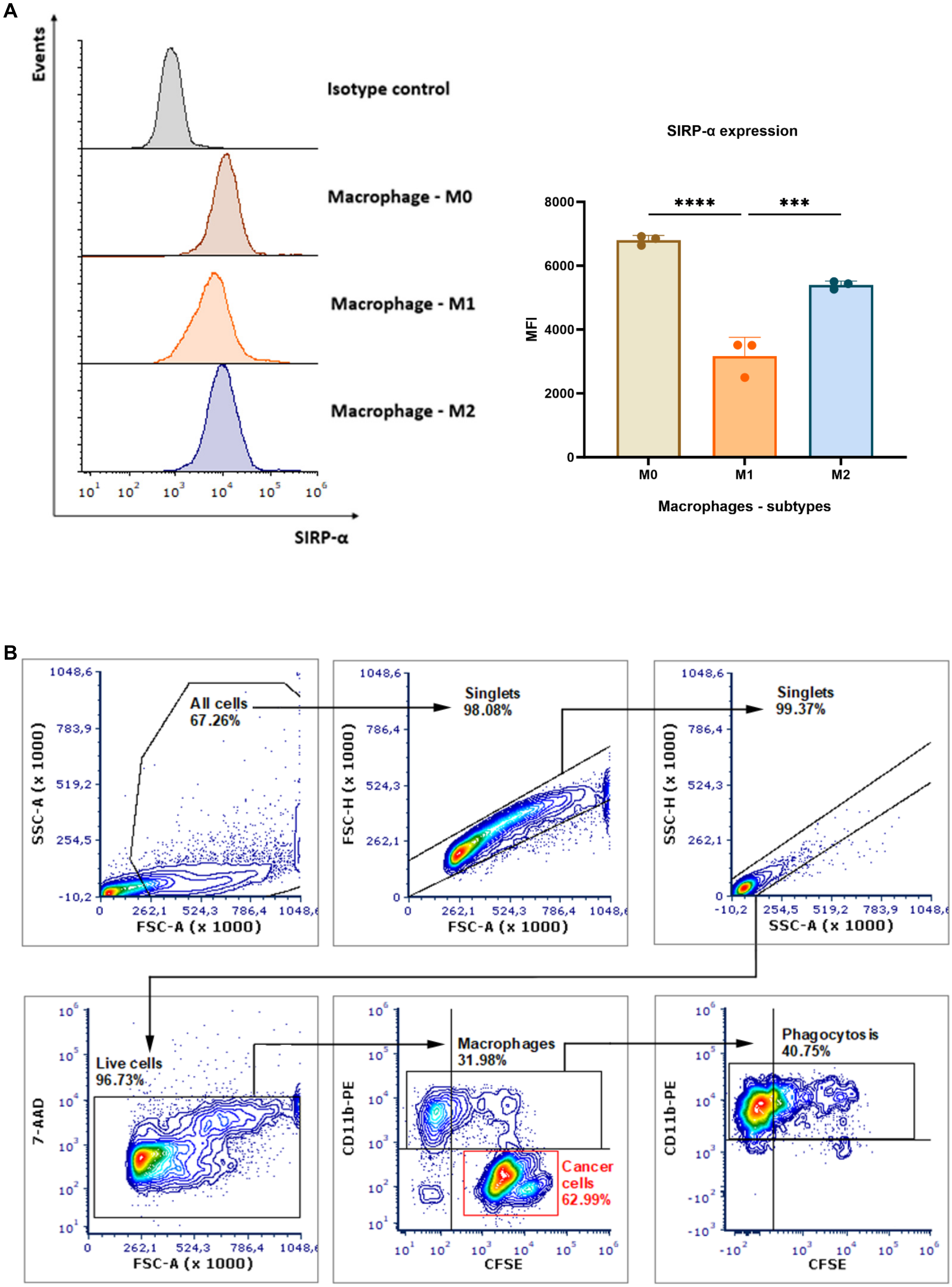
A
Events
Isotype control
Macrophage - M0
Macrophage - M1
Macrophage - M2
SIRP-α
SIRP-α expression
MFI
****
***
M0
M1
M2
Macrophages - subtypes
B
All cells
67.26%
Singlets
98.08%
Singlets
99.37%
SSC-A (x 1000)
FSC-A (x 1000)
FSC-H (x 1000)
SSC-H (x 1000)
Live cells
96.73%
7-AAD
Macrophages
31.98%
Cancer
cells
62.99%
CD11b-PE
CFSE
Phagocytosis
40.75%

Figure S5

Figure S5

SIRP-alpha expression in Tumor Associated Macrophages (TAMs) and gate strategy for co-culture assay. A) Representative histogram and histogram bars showing that SIRP-alpha is constitutively expressed in different macrophage phenotypes, with higher expression in M0 and M2-like compared to M1-like macrophages (adapted from *Aroldi et al, J Cell Mol Med 2023*; one-way ANOVA with multiple comparisons correction; SIRP-α $F_{(2,6)} = 80.21$; experimental triplicate, one representative donor; ***$p < 0.001$, ****$p < 0.0001$). **B)** To define phagocytosis, gate strategy for co-culture analysis was performed by ruling out debris and dead cells through morphology and 7-AAD staining, together with doublet removal. Phagocytosis was counted as percentage based on the number of 7-AAD/$CD11b^+$/$CFSE^+$ events out of all 7-AAD^-/$CD11b^+$ events (total number of macrophages). Depicted flow cytometry plots were representative of at least 10 experimental replicates and showed co-culture assay with donor-derived macrophages and SUP-M2 cell line (condition after administration of anti-CD47 mAb, previously exposed to crizotinib 0.5 µM for 20 hours).

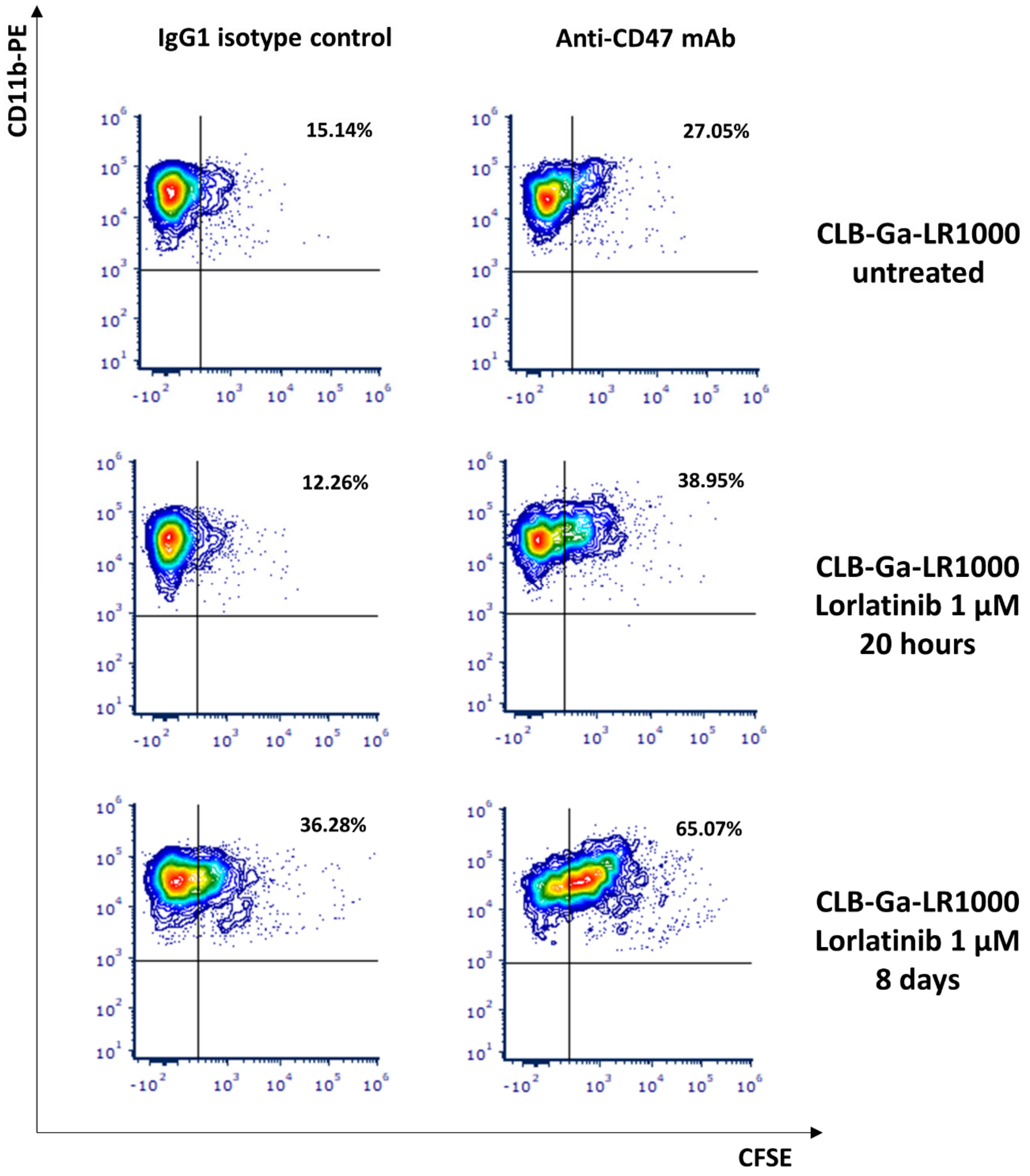

Figure S6

Figure S6. Phagocytosis assay of representative ALK-positive cancer cell line, treated with anti-CD47 mAb, after exposure to ALKi. Representative flow cytometry plot comparison for one ALK-positive cancer cell line (i.e., resistant Neuroblastoma cell line, CLB-Ga-LR1000) in terms of phagocytosis, with “IgG1 isotype control” and “anti-CD47 mAb” (left and right column, respectively), after exposure to lorlatinib 1 µM at two different timepoints (20 hours, 8 days).

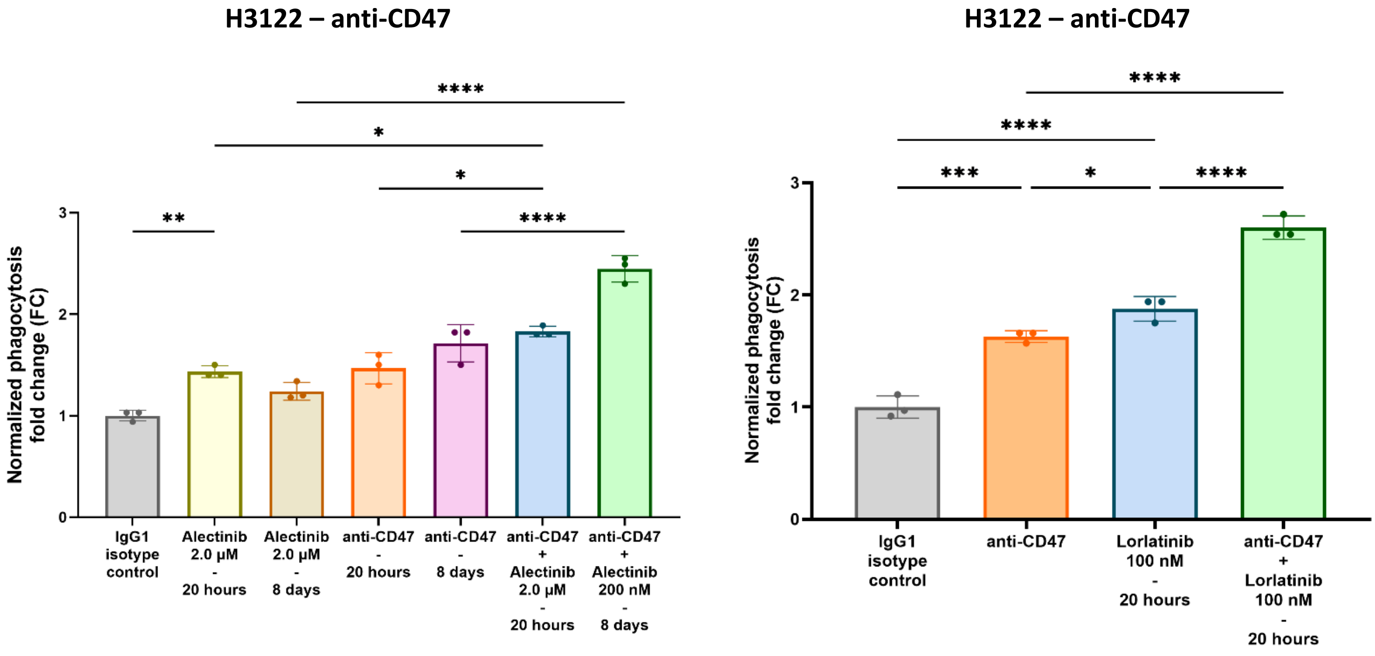

Figure S7

Figure S7. Analysis of increased phagocytosis in TKIs-sensitive ALK-positive NSCLC cell lines, treated with anti-CD47 mAb, and previously exposed to ALKi. Histogram analysis of phagocytic rate in TKIs-sensitive ALK-positive NSCLC cell line (H3122), previously exposed to alectinib or lorlatinib, treated with anti-CD47 mAb (left panel: alectinib 2 µM for 20 hours and 200 nM for 8 days; right panel: lorlatinib 100 nM for 20 hours; H3122.S. One-way ANOVA with multiple comparisons correction; H3122$_{\text{ALECTINIB}}$ $F_{(3,8)} = 87.27$, H3122$_{\text{LORLATINIB}}$ $F_{(3,8)} = 149.0$; experimental triplicates, $n = 3$ donors; * $p < 0.05$, ** $p < 0.01$, *** $p < 0.001$, **** $p < 0.0001$).

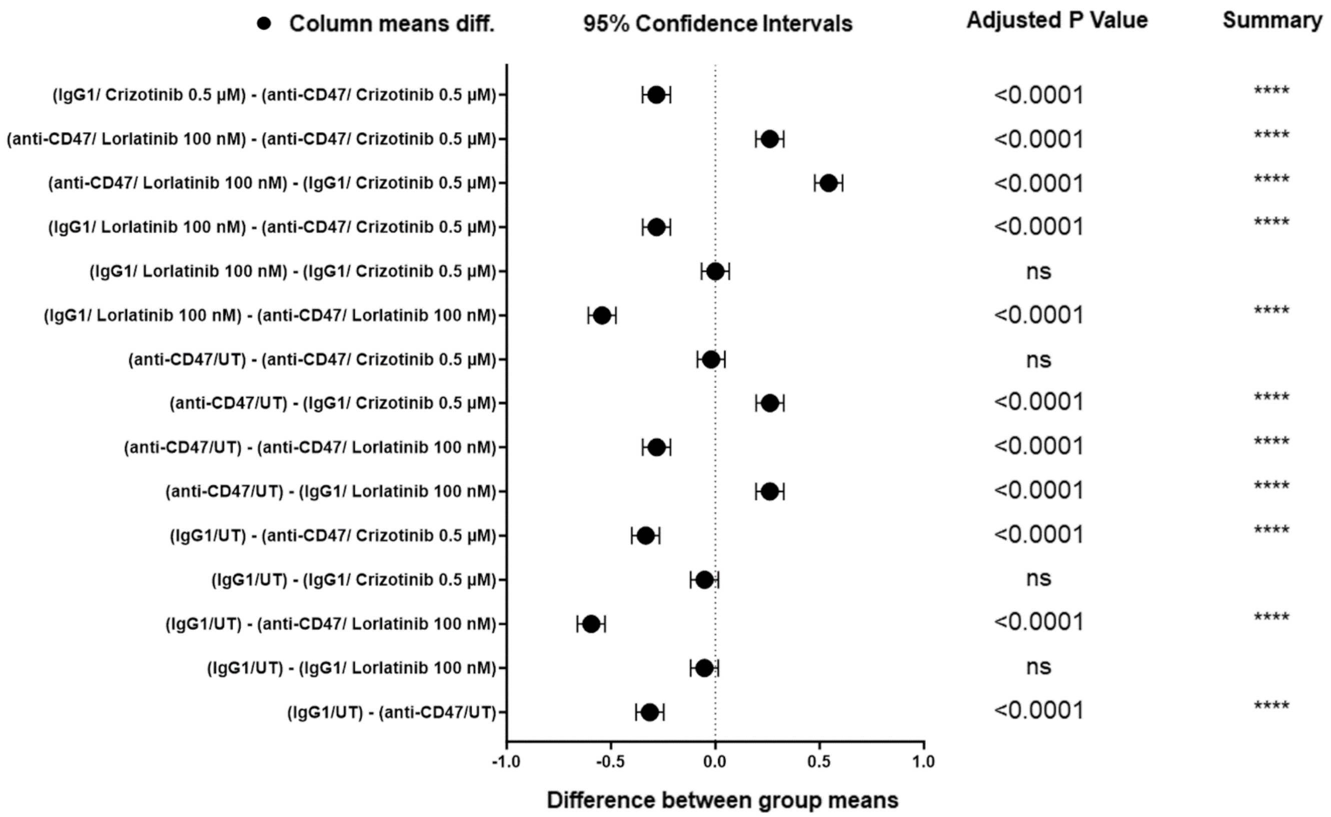

Figure S8

Figure S8

Mean 95% Confidence Interval (95% CI) and adjusted *p* value from Tukey's multiple comparison test for AS4 cell line co-culture analysis by fluorescent microscopy. Recap of multiple comparison tests (Tukey's method), corresponding 95% CI and adjusted *p* value of the conditions developed after co-culture assays for AS4 cell line; negative values of differences between group means express statistical significance (one-way ANOVA with multiple comparisons correction; AS4 $F_{(5,12)} = 275.6$; technical triplicate, one representative donor; ns: not significant; **** $p < 0.0001$).
